# Extensive eczema herpeticum in a previously well child

**DOI:** 10.1186/s12245-022-00425-5

**Published:** 2022-05-21

**Authors:** Manal Almoalem, Ibrahim AlAlharith, Hussa Alomer, Azzam Almarri, Awadh Alyami, Rakan Hamzah, Othub Albalawi, Salwa Alnoaimi

**Affiliations:** 1 Department of Pediatrics, Salmaniya Medical Complex, Manama, Bahrain; 2 College of Medicine & Medical Science, Arabian Gulf University, Manama, Bahrain

**Keywords:** Acyclovir, Eczema herpeticum, Herpes simplex virus, Rash, Case report

## Abstract

**Background:**

Eczema herpeticum, also known as Kaposi varicelliform eruption, is a potentially life-threatening disseminated cutaneous viral infection. In the majority of cases, this condition develops as a complication in patients with atopic dermatitis. However, it may arise in a wide spectrum of pre-existing skin conditions, including psoriasis, seborrheic dermatitis, contact dermatitis, cutaneous T cell lymphoma, pemphigus vulgaris, and others.

**Case presentation:**

We present the case of a 2-year-old boy who was brought to the emergency department because of a high-grade fever and rash. The fever started 2 days before his presentation, and its maximum measurement was 39.6°C. The following day, the patient developed numerous painful, pruritic vesiculopustular eruptions, and oozing involving the lips, rendering the patient unable to tolerate oral feeding. The patient was seen by the dermatology team who diagnosed the child as having eczema herpeticum. The patient was commenced on antiviral and empirical antibiotic therapy in the form of intravenous acyclovir and cephalexin along with topical fusidic acid and panthenol. The patient showed clinical improvement with resolution of the fever and partial involution of the rash 2 days following the administration of the antimicrobial therapy.

**Conclusion:**

Eczema herpeticum is a rare clinical entity that can result in significant morbidity. The case highlights the importance of considering the diagnosis of eczema herpeticum in the appropriate clinical settings, even in patients who were not known to have any prior skin disorder.

## Background

Eczema herpeticum, also known as Kaposi varicelliform eruption, is a potentially life-threatening disseminated cutaneous viral infection. It is typically caused by the herpes simplex virus, but it can develop by coxsackie, vaccinia, and smallpox viruses. In the majority of cases, this condition develops as a complication in patients with atopic dermatitis [1]. However, it may arise in a wide spectrum of pre-existing skin conditions, including psoriasis, seborrheic dermatitis, contact dermatitis, cutaneous T cell lymphoma, pemphigus vulgaris, and others [2]. Here, we present the case of a previously healthy boy with extensive eczema herpeticum.

## Case presentation

We present the case of a 2-year-old boy who was brought to the emergency department by his parents because of a high-grade fever and rash. The fever started 2 days before his presentation, and its maximum measurement was 39.6°C. The parents reported partial resolution of the fever with paracetamol. The following day, the patient developed numerous painful, pruritic vesiculopustular eruptions and oozing involving the lips, rendering the patient unable to tolerate oral feeding. On the same day, the rash spread sequentially spread to involve the face, neck, and upper trunk. The genitalia was spared. There was no history of upper respiratory tract symptoms, abdominal pain, or change in urinary or bowel habits. The patient had no sick contact and was fully up to date with his vaccination schedule. The patient was previously healthy with unremarkable past medical history.

**Fig. 1:**
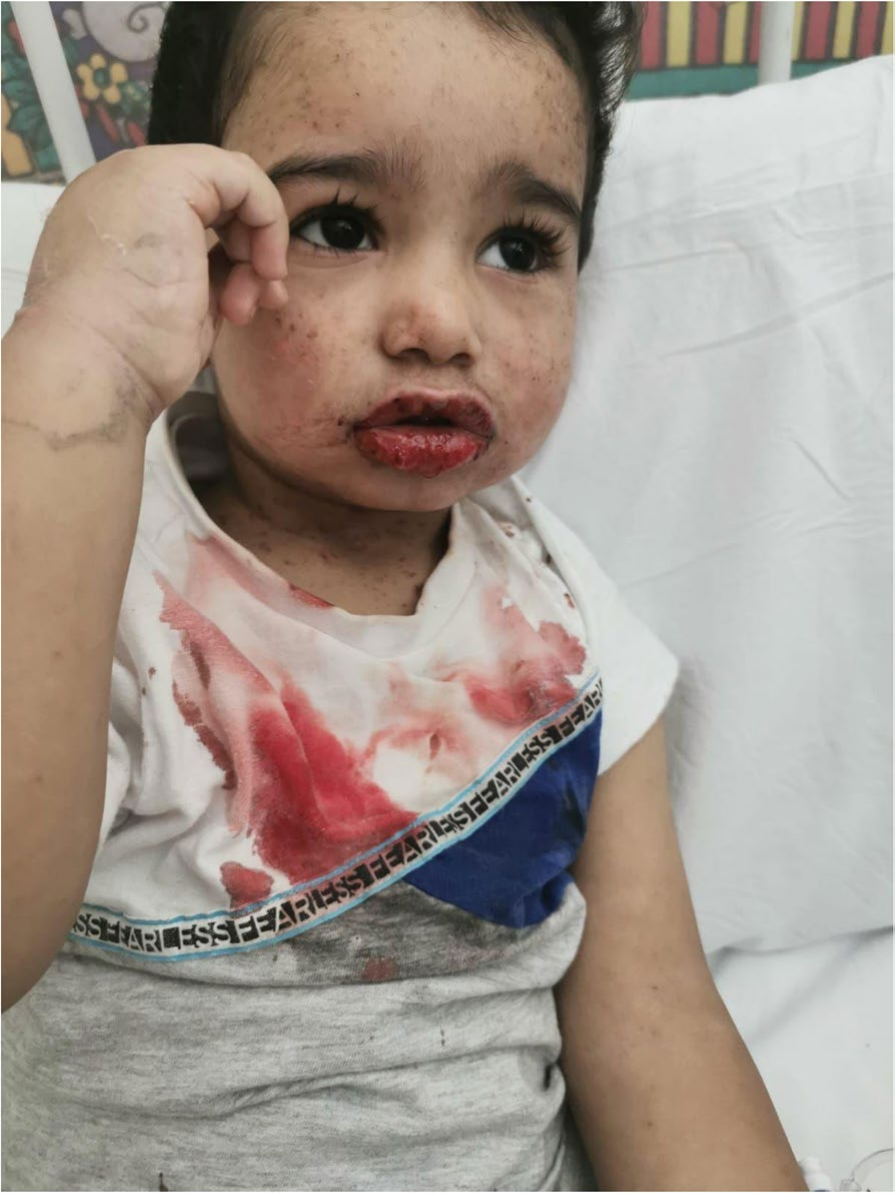
Photograph of the child showing severely cracked oozing lips

**Fig. 2:**
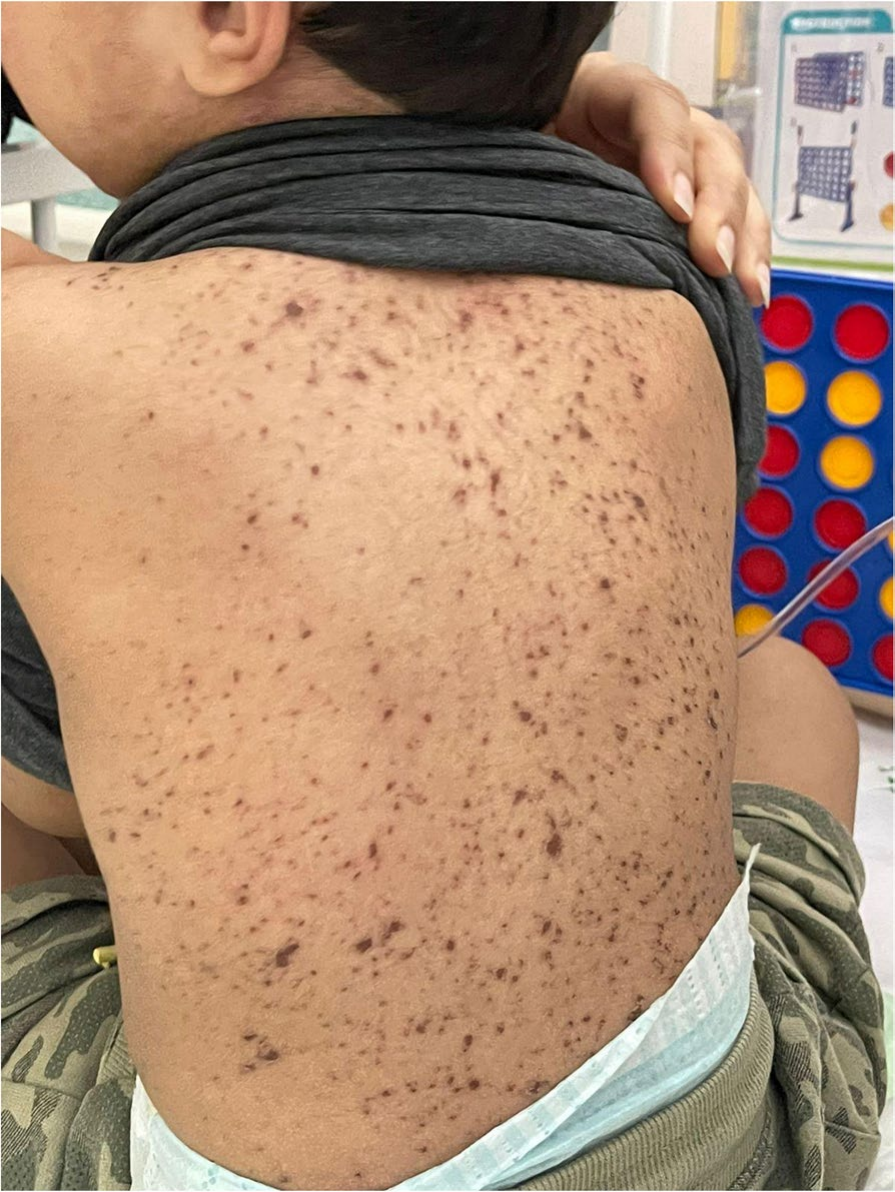
Photograph of the child showing the disseminated non-blanching maculopapular rash involving the back

Upon examination, the child appeared sick with severely cracked oozing lips (Fig. 1). Further, disseminated non-blanching maculopapular rash involving the face and trunk was observed (Fig. 2). His vital signs were as follows: a pulse rate of 118 bpm, a blood pressure of 124/80 mmHg, a respiratory rate of 24 bpm, and a temperature of 39°C. Head and neck examination showed erythematous congested pharynx with palpable cervical lymph nodes. Cardiorespiratory and abdominal examinations revealed normal findings. Initial laboratory investigation revealed a hemoglobin level of12.1 g/dL, leukocyte count of 8000/μL, a platelet count of 455,000/μL, and C-reactive protein of 37.8 nmol/L. A swab culture of the lesions revealed no bacterial growth. Chest radiograph demonstrated clear lung fields with no airspace opacity.

Given the inability to tolerate oral feeding, the patient was admitted for further evaluation and management. The patient was seen by the dermatology team who diagnosed the child as having eczema herpeticum. The patient was commenced on antiviral and empirical antibiotic therapy in the form of intravenous acyclovir and cephalexin along with topical fusidic acid and panthenol. The patient showed clinical improvement with resolution of the fever and partial involution of the rash 2 days following the administration of the antimicrobial therapy. The patient started to tolerate oral feeding. The patient was discharged after 6 days of admission and was prescribed symptomatic treatment. In the follow-up visit after 2 weeks, the patient had complete resolution of the rash with no active complaints.

## Discussion

We presented a rare case of eczema herpeticum in a child who was not known to have any dermatological conditions. This condition was first described by Moriz Kaposi in 1887 [3]. The exact pathogenesis of eczema herpeticum remains unclear. It is suggested that a complex interplay of epidermal barrier breakdown with impairment in the cell-mediated and humoral immunity [4]. In the present case, however, the patient did not have any history suggesting immunodeficiency or previous skin disorders. While eczema herpeticum can affect both genders, a prior study found that male patients experience a more severe course of the disease [5].

The diagnosis of eczema herpeticum is made mainly by the clinical findings of disseminated vesiculopustular rash, which can evolve into hemorrhagic vesicles. Systemic symptoms, including fever, may occur in over 50% of patients [5]. Tzank smear can be performed which can demonstrate multinucleated giant cells on the Wright-Giemsa stain. Direct fluorescent antibody staining, polymerase chain reaction, and viral cultures can determine the causative viral agent [6]. Such investigations were not performed in the present case and the diagnosis of eczema herpeticum was made solely on the clinical grounds that supported the rapid clinical improvement following the administration of acyclovir.

Acyclovir is the treatment of choice for eczema herpeticum. Considering the low bioavailability of oral acyclovir, its use should be limited to mild cases. The management should be started promptly as delayed acyclovir administration was found to prolong the need for hospital stay [7]. Empiric antibiotic therapy was not found to lower the hospital stay or the mortality rate [8]. We initiated the antibiotic therapy considering the extensive involvement of the skin while the result of the bacterial swab culture was not available. Notably, superinfection with *Staphylococcus aureus* in patients with eczema herpeticum may reach up to 30% [7].

Eczema herpeticum can be a cause of significant morbidity and mortality if its diagnosis and management were delayed. Prior to the use of acyclovir, the mortality rate from eczema herpeticum reaches 50% [9]. Further, the complications of eczema herpeticum can include meningoencephalitis, keratoconjunctivitis, and disseminated intravascular coagulation [10, 11].

## Conclusion

Eczema herpeticum is a rare clinical entity that can result in significant morbidity. The case highlights the importance of considering the diagnosis of eczema herpeticum in the appropriate clinical settings, even in patients who were not known to have any prior skin disorder. Prompt administration of acyclovir is imperative to shorten the disease course and prevent its dissemination.

## Data Availability

Not applicable
