# Correction: Extensive eczema herpeticum in a previously well child

**DOI:** 10.1186/s12245-022-00428-2

**Published:** 2022-06-01

**Authors:** Manal Almoalem, Ibrahim AlAlhareth, Hussa Alomer, Azzam Almarri, Awadh Alyami, Rakan Hamzah, Othub Albalawi, Salwa Alnoaimi

**Affiliations:** 1 Department of Pediatrics, Salmaniya Medical Complex, Manama, Bahrain; 2 College of Medicine & Medical Science, Arabian Gulf University, Manama, Bahrain

**Correction: Int J Emerg Med 15, 21 (2022)**

**https://doi.org/10.1186/s12245-022-00425-5**

Following publication of the original article [1], the authors identified an error in the author name of Ibrahim AlAlhareth.

The incorrect author name is: Ibrahim AlAlharith

The correct author name is: Ibrahim AlAlhareth

The author group has been updated above and the original article [1] has been corrected.
